# A Highly Sensitive Two-Dimensional Inclinometer Based on Two Etched Chirped-Fiber-Grating Arrays †

**DOI:** 10.3390/s17122922

**Published:** 2017-12-15

**Authors:** Hung-Ying Chang, Yu-Chung Chang, Wen-Fung Liu

**Affiliations:** 1 Program of Electrical and Communications Engineering, Feng-Chia University, 100 Wenhwa Rd., Seatwen, Taichung 40724, Taiwan; hungying.chang@gmail.com; 2 Department of Electrical Engineering, National Changhua University of Education, 2 Shida Rd., Changhua 50074, Taiwan; 3 Department of Electrical Engineering, Feng-Chia University, 100 Wenhwa Rd., Seatwen, Taichung 40724, Taiwan; wfliu@fcu.edu.tw

**Keywords:** etched fiber Bragg grating, chirped fiber Bragg grating (CFBG), level meter, inclinometer, tilt meter, fiber sensor

## Abstract

We present a novel two-dimensional fiber-optic inclinometer with high sensitivity by crisscrossing two etched chirped fiber Bragg gratings (CFBG) arrays. Each array is composed of two symmetrically-arranged CFBGs. By etching away most of the claddings of the CFBGs to expose the evanescent wave, the reflection spectra are highly sensitive to the surrounding index change. When we immerse only part of the CFBG in liquid, the effective index difference induces a superposition peak in the refection spectrum. By interrogating the peak wavelengths of the CFBGs, we can deduce the tilt angle and direction simultaneously. The inclinometer has a resolution of 0.003° in tilt angle measurement and 0.00187 rad in tilt direction measurement. Due to the unique sensing mechanism, the sensor is temperature insensitive. This sensor can be useful in long term continuous monitoring of inclination or in real-time feedback control of tilt angles, especially in harsh environments with violent temperature variation.

## 1. Introduction

Due to the increased requirements on the accuracy of contemporary civil engineering and mechanical control, high-precision two-dimensional inclinometers are a necessity in modern industry. Precise measurement of the tilt angle and direction is very important in many fields, such as in level determination for buildings or instrumentation, mechanical alignment, aircraft control, satellite antenna positioning, motion control of robots, etc. There are many conventional methods for tilt angle and direction measurements using electrical, ultrasonic, mechanical, and optical techniques. Among these methods, optical fiber grating based sensors have been found ideal in many applications due to their intrinsic advantages of small size, high sensitivity, long-term stability multiplexing capability, and immunity to electromagnetic interference (EMI), which make them suitable in field-deployed monitoring [1,2]. In recent years, several fiber Bragg grating (FBG)-based inclinometer or tilt sensors with different configurations for one or two-dimension (1-D or 2-D) measurements have been reported [3,4,5,6,7,8,9,10,11,12,13,14,15]. For example, Dong et al. have proposed a design based on three FBGs. Each FBG is fixed on the top surface of a steel flake to form an inverted pendulum. The accuracy of the measured tilt angle is ±0.13° with a resolution of 0.02° [3]. Ni et al. reported a tilt sensor with an improved sensitivity of 0.009° based on connecting four FBGs to a mass to form an inverted tetrahedron. As the sensor tilt, the tensions on the four FBGs change and sensitive measurement can be achieved [4]. In [5,6], Bao et al. used a similar operating principle as in [4], but with different FBG numbers and structures. A tilt angle detection range of ±40° and a sensitivity of 0.096 nm/degree were demonstrated. Although the tile angle detection range was improved, the sensitivity was compromised. All of these designs relied on the swing of the mass when tilt the sensors. As the mass is hung directly from the fiber, any unknown resonance leads to a random vibration of the pendulum system. In addition, this kind of design inevitably renders a large sensor size. 

Optical fiber based sensors are not only immune from EMI, but also not susceptible to humidity or corrosive damage as in electronic sensors. The typical resolution of conventional electronic inclinometer is on the order of 0.01°. It was desirable to have higher sensitivity in a wide detection range. In our recently studies, we proposed a novel detection mechanism based on etched chirped fiber Bragg grating (CFBG). It is known that it is possible to greatly improve the sensing sensitivity of a FBG by removing most of the cladding layer to expose the evanescent wave to the surrounding medium [16]. There have been demonstrated inclinometers based on etched fiber grating [17], but the resolution is not improved compared to conventional methods. We recently demonstrated a novel etched-CFBG based sensing mechanism with superior sensitivity. By putting part of the etched chirped grating in liquid, the effective refractive index of this part is varied, which results in a shift in its reflection spectrum, while the reflection spectrum from the other part in air remains unchanged. With proper arrangement, the reflected spectra from the two parts would overlap to form a superposition peak in the measured spectrum. When the air-liquid boundary moves along the grating, the superposition peak shifts accordingly. This is a truly novel way of fiber Bragg grating based sensing and is first demonstrated by our group. We have demonstrated using this method as a liquid level indicator with a sub-10 μm resolution [18]. Based on the high accuracy in liquid-level measurement, we further extend its application to a two-dimensional (2D) inclinometer by installing two etched CFBG in the inner wall of a cylindrical container separated from each other by 90° in the azimuth direction [19]. By examining the peak wavelengths and relative peak positions, we can deduce the tilt angle and direction of tilting. The detection range of the tilt angle is 0–35° and the directional angle is 0–360°, respectively. The sensitivity for the tilt angle is 0.303 nm/degree, which corresponds to a resolution of 0.033° when acquired by an optical spectrum analyzer with a resolution of 0.01 nm. 

Here we propose a disk-shaped 2-D inclinometer with a totally different configuration, which not only greatly reduces the sensor footprint but also greatly improves the dynamic range and sensitivity. Particularly, the sensor design is readily compatible with typical inclinometer or level meter, so it can be applied intuitively. The present inclinometer has a resolution of 0.003° for tilt angle measurement, which is about one order improvement compared to the previous design. The sensor configuration is detailed in the next section. Briefly, the container is partially filled with liquid and air as is in a typical level meter. The sensor contains four etched CFBGs fixed on the curved top cover of the plate-shaped container. When the air bubble moves freely in the container, the superposition peaks of the four CFBGs move accordingly. By examining the peak wavelengths and relative positions, we can deduce the tilt angle and direction simultaneously in real-time. In addition, this sensor is highly flexible in its detection range and sensitivity, which can be modified by adjusting the geometry of the container and the arrangement of the CFBGs. 

For many applications, it is desirable to have temperature-insensitive sensors. There are many FBG based temperature-insensitive tilt sensors or inclinometers [7,8,9,10,11,14]. However, these sensors are either large in size or lack high sensitivity. We have demonstrated that because the sensing mechanism is based on measuring the peak shift of the overlapped spectra, the thermal or strain effect on the FBG is compensated. The superposition peak shift has a very low temperature dependence of 0.008 nm/°C [18,19]. For the present sensor, because the tilt angle is determined by subtracting the wavelengths of the two peaks on the same array, the temperature effect is further eliminated. Therefore, this sensor provides temperature-insensitive measurement on inclination, which is highly desirable in many applications, especially for long-term monitoring of environments with violent temperature surges.

In addition, this inclinometer has a real-time sensing capability, which is critical for security monitoring systems, such as construction structure surveillance or geological environment monitoring for emergency alerts. A small-sized design not only makes the sensor suitable for embedded multiplexing monitoring but also robust for field-deployable measurements in harsh environments. The sensor design is completely compatible with conventional sensors and is readily applicable to ongoing applications.

## 2. Principle and Sensor Configuration

A CFBG is a type of fiber Bragg grating, which was made by axially varying either the grating period (Λ) or the core effective refractive index (*n_eff_*). In this study, the CFBGs were fabricated by exposing a 248 nm laser beam from a KrF excimer laser through a chirped phase mask on standard single mode fibers. The chirped phase mask used in this paper has a linear increment in the grating period with a chirped rate of 9.08 nm/cm. The Bragg reflection wavelength at the axial location *z* along the fiber core of the CFBG can be expressed as [20,21]:

(1) $$\lambda (z)=2n_{eff}(\Lambda_{0}+\frac{C}{2n_{eff}}z)$$

where Λ_0_ is the initial grating period, which corresponds to the smallest reflection wavelength of the CFBG, *n_eff_* denotes the effective index, and *C* = d*λ*/d*z* is the chirp rate. A CFBG can be regarded as a grating structure made up of a series of short Bragg gratings with increasing period.

**Figure 1:**
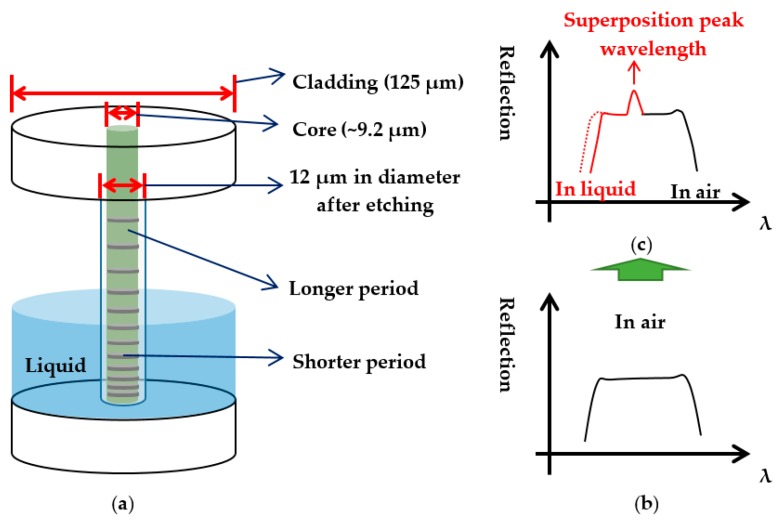
(**a**) Illustration of the etched chirped fiber grating and the sensing principle; (**b**) Illustration of reflection spectrum of the etched CFBG in air; and (**c**) the shorter period part of the grating immersed in liquid.

The CFBG used for this study was fabricated on a standard SMF-28 (Fujikura Corp., Tokyo, Japan) with a core diameter of 9.2 μm. The cladding layer of CFBG was etched off from the original 125 μm to 12 μm in diameter by a 20% HF solution. Figure 1 shows the sensor configuration in which the shorter grating period part was immersed in the liquid for level measurements. For increasing the mechanical strength of the sensing head, the CFBG was glued on a Teflon holder. After etching, the CFBG was not moved out of the holder and the whole apparatus was immersed in water for the following measurements [18].

In this study, we etched the chirped fiber gratings to expose the evanescent field to the surrounding medium to improve the sensing sensitivity. In the etching process, an optical spectrum analyzer (OSA) was used to monitor the fiber diameter. When the grating reflection wavelength was shifted to the shorter side, it indicated that the cladding layer had been etched thin enough and the diameter of the fiber was about 12 μm. Meanwhile, the effective index was strongly affected by the surrounding medium. The effective index change induced shift of the grating wavelength *λ_CFBG_* can be expressed as:

(2) $$\lambda_{CFBG}=2(n_{eff}\Lambda_{0}+\Delta n\Lambda_{0})$$

where Δ*n* is the effective index variation due to the surrounding medium.

When the shorter period part of the etched CFBG is immersed in the liquid, the increased effective index causes the reflection spectrum of this part to redshift. Therefore, a portion of the spectrum of the immersed grating overlaps with the spectrum of the longer period CFBG. The overlapped spectra result in a superposition-spectrum peak, as illustrated in Figure 1c. This superposition-spectrum peak wavelength *λ_p_* is a function of liquid level *z* and can be expressed as [18]:

(3) $$\lambda_{p}=2n_{eff}\Lambda_{0}+Cz+\Delta n\Lambda_{0}$$

where *n_eff_* is the grating effective index, Λ_0_ is the grating initial period, *C* is the chirp rate, *z* is the position of liquid level, and Δ*n*Λ_0_ is the variation of reflection wavelength.

In order to use this method to detect the liquid level variation, one can simply take a derivative of Equation (3) as:

(4) $$\Delta \lambda_{p}=C\times \Delta z$$

where Δ*λ_p_* is the wavelength shift of the overlapped peak and Δ*z* is the variation of liquid level.

**Figure 2:**
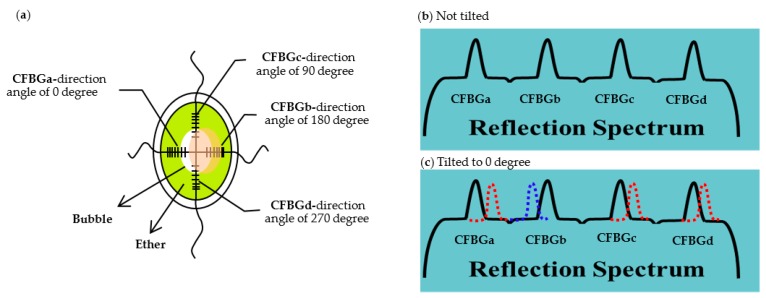
(**a**) The schematic diagram of the fiber-optic inclinometer. Illustrations of the reflection spectra when the sensor is (**b**) not tilted and (**c**) tilted to 0°.

In this study, two etched CFBGs arrays are cross-wisely installed in a plate-shaped container as the sensing elements of the inclinometer as shown in Figure 2a. Each fiber has two inscribed CFBGs. The length of each chirped fiber grating is 7 mm. The separation between two chirped fiber gratings is 6 mm with the longer period part facing the center of the sensor. Diethyl-ether (ethoxyethane, or simply ether) and an air bubble are filled in the container. The inner diameter of the plate is 20 mm and the diameter of the bubble is 10 mm. Therefore, the air-liquid boundaries of the bubble are located at the middle of the CFBGs as shown in Figure 2. The structure is similar to a typical level meter. To avoid saturation of the superposition of the peaks, the reflectivity of the CFBGs is designed as 40%. The tilt angle and direction can thus be monitored through the peak wavelength shifts of the four CFBGs. Figure 2b,c illustrate the relative change of the superposition peaks at different situations. When the sensor is not tilted, the four peaks are located at their center positions as shown in Figure 2b. When the sensor is tilted to 0°, due to the movement of the air bubble, as illustrated in Figure 2a, for CFBGa, the liquid-air boundary shifts to the longer period grating side, which results in a red shift for the peak of CFBGa. Similarly, for CFBGb, the liquid-air boundary shifts to the shorter part and results in blue shift of the peak. For CFBGc and CFBGd, due to the movement of the air bubble, the liquid-air boundaries both move to the longer period part and cause the peaks to redshift, but not as much as CFBGa.

## 3. Experimental Results and Discussions

**Figure 3:**
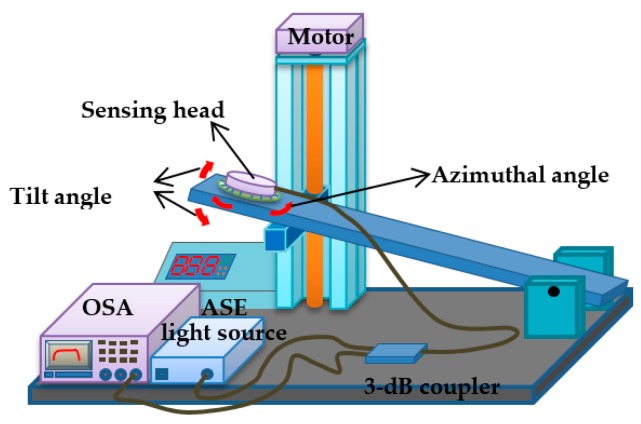
The experimental setup for evaluating the proposed 2-D inclinometer. The tilt angle can be controlled by the slanting stage. The titling direction is controlled by rotating the azimuthal angle of the plate-shaped sensor.

The setup that we used to evaluate the proposed two-dimensional fiber-optic inclinometer is illustrated in Figure 3. The tilt angle is controlled by slanting the electrical platform. The tilting direction (directional angle) was controlled by rotating the azimuthal angle of the sensor head. The range of tilt angle was controlled between 0° and 2.5°, and the range of directional angle was from 0 to 360°. Two etched CFBG arrays were connected to the optical spectrum analyzer (OSA, Q8384, Advantest Corp., Tokyo, Japan) and a wide-band amplified-spontaneous-emission (ASE, FL7002, Thorlabs Corp., Newton, NJ, USA) light source through a 3-dB coupler for real-time spectrum analysis. The acquired spectra by the OSA were sent to a computer for further analysis.

**Figure 4:**
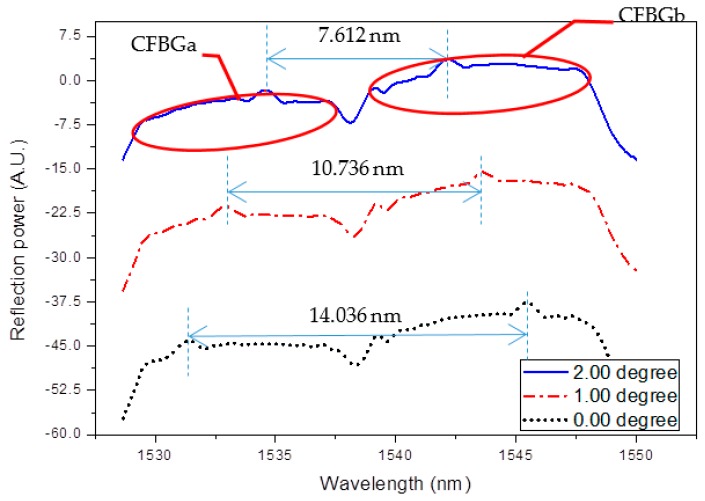
Reflection spectra of the CFBGa and CFBGb array at 0, 1, and 2° tilt angles at the directional angle of 0°. The spectra are shifted in the *y*-axis for presentation. The double-sided arrows indicate the separation between the superposition peaks of CFBGa and CFBGb.

When the tilt angle or directional angle was changed, the lengths of the CFBGs immersed in the ether were also changed. Thus, the tilt angle and the tilt direction could be obtained by measuring the peak wavelength shifts of the four CFBGs. Figure 4 shows reflection spectra of the CFBGa and CFBGb array at 0, 1, and 2° tilt angles at the directional angle of 0°. The spectra are shifted in the *y*-axis to clearly show the variation in the relative positions of the superposition peaks. The bandwidth of CFBGa is from 1529.54 to 1537.10 nm, and the bandwidth of CFBGb is from 1539.12 to 1547.53 nm. As seen in the figure, when the tilt angle increases, the superposition peak wavelength of CFBGa shifts toward a longer wavelength, while the peak of CFBGb shifts toward a shorter wavelength. The peak wavelength is determined as the local maximum data point in the spectrum. Therefore, the tilt angle can be obtained by measuring the relative wavelength shifts of the two peaks. Since the two peaks both shift when tilted, the larger dynamic range greatly improves the sensor resolution when compared to using a single peak for measurement as in our previous version [19]. The sensitivity for tilt angle measurement at the directional angle of 0° is 3.3 nm/°, which corresponds to a resolution of 0.003° when measured with an OSA with a resolution of 0.01 nm, which is about one order of magnitude improvement compared to the previous version. Due to the unique sensing mechanism, the superposition peak shift already has a very low temperature dependence of 0.008 nm/°C due to the compensated thermal and strain effects on the FBG as can be seen from Equation (3) [19,20]. Here, because the tilt angle is obtained by subtracting the wavelengths of the two peaks, the temperature has the same effect on both CFBGs, after subtracting, the temperature effect is eliminated. Therefore, the sensing mechanism for the proposed inclinometer is temperature insensitive, which is highly desirable in many applications, especially in environments where the temperature changes violently. 

**Figure 5:**
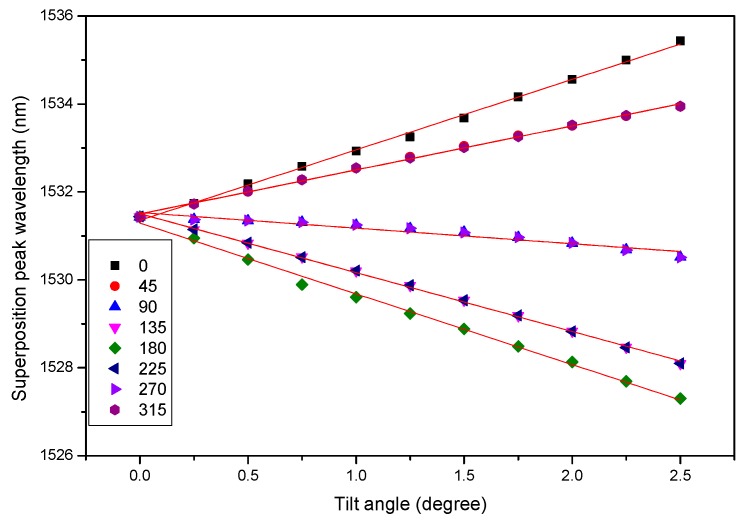
The relation between the peak wavelength of CFBGa and the tilt angle at different directional angles (0°–315°). Different directional angles are indicated with different label shapes and colors as marked in the figure.

Figure 5 shows the relation between the peak wavelengths of CFBGa and the tilt angles when the sensor is tilted at different directional angles, respectively. By examining Figure 4 and Figure 5, it is obvious that the tilt angle and direction can be simultaneously obtained by the permutations of the red-shifted and blue-shifted peaks of the two CFBGs arrays. In Figure 5, it is clear that when the tilt direction is at 0°, we have a positive maximum wavelength shift, while the negative maximum wavelength shift occurs at a tilt direction of 180°. This is expected as in the two directions the displacements of the air bubble are the largest, because the CFBGa-CFBGb array is installed on the 0°–180° axis of the disk-shaped sensor, as shown in Figure 2. Therefore, the relationship curves of CFBGb are similar to those of CFBGa, except the order of the directional angles are reversed. Similarly, the relationship curves of CFBGc are almost identical to those of Figure 5 except for a directional angle difference of 90° because the CFBGc is installed orthogonally to CFBGa. Thus, the maximum wavelength shifts are located at 90° and 270° directional angles, respectively, for CFBGc, and CFBGd has a relation symmetric to CFBGc. The knowledge about the relations between the peak wavelengths and tilt angles and directions allows us to compute the tilt angle and direction simultaneously in real-time. The sensor, therefore, enables real-time feedback control of the tilt angle and direction, which can be useful in precision robotic control.

**Figure 6:**
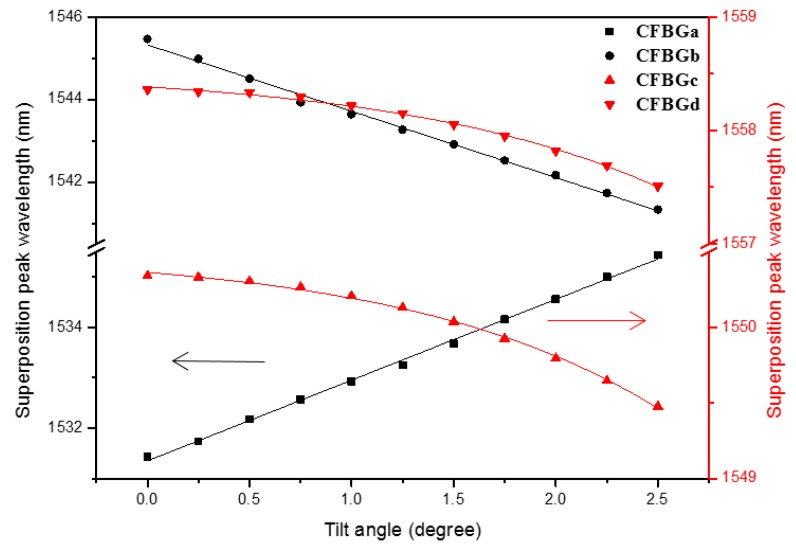
The relation between the peak wavelengths of four CFBGs and the tilt angle at the direction angle of 0°. The black points belong to the ordinate on the left and the red points belong to the ordinate on the right. The arrows help to identify the ordinate the data points belong to.

In order to understand how the superposition peaks of the four CFBGs shift as the sensor tilts, we plot the relations between the peak wavelengths and the tilt angles, as shown in Figure 6. The tilt direction is 0° for this plot. The points for CFBGa and CFBGb are labeled in black and the points for CFBGc and CFBGd are red. The two CFBG arrays are inscribed at distinct wavelength ranges for easier reading. In order to put the data points from the two CFBG arrays on the same plot to illustrate the shifts of the peaks as the sensor tilt, we overlapped the distinct wavelength ranges in Figure 6. The black labels are referred to the ordinate on the left, while the red ones are referred to the right ordinate. As seen in the figure, as the tilt angle increases, only the peak wavelength of CFBGa increases accordingly, while the other three CFBGs decrease with different tendencies. CFBGa and CFBGb have the same amount of wavelength shifts, but with different signs. CFBGc and CFBGd do not have a linear relationship between the peak wavelength change and the tilt angle. This is because the peak wavelength variation is due to the air-liquid boundary movement along the grating. The change of the peak wavelength for CFBGc and CFBGd is due to the shape of the circular circumference of the air bubble as shown in Figure 2. The two CFBG arrays are crisscrossed, placed in the container with each CFBG separated by 90°. The reason for similar wavelength shifts, but toward opposite sides for CFBGa and CFBGb, is because they are connected in series and the chirping is symmetric with a longer period connected to each other. When the sensor is tilted to 0°, the air bubble moves toward the direction of 180° and the amount of the liquid level change on CFBGa and CFBGb are the same. As the air bubble moves, the CFBGa immerses more into the liquid, which causes the superposition peak to shift toward the longer side. In contrast, as the bubble moved toward CFBGb, less grating is immersed in the liquid, resulting in the opposite peak shift. On the other hand, the relation between the superposition peak wavelength shift and the tilt angle is a parabolic line for CFBGc and CFBGd. This is due to the circular circumference of the air bubble as it moves along the gratings. The distinct tendencies among the gratings make it straightforward for us to determine the tilt direction.

From the above discussion, it is obvious that the relation between the peak wavelengths of the four CFBGs and the tilt and directional angles is a 1-to-1 correspondence. With the measured peak wavelengths, we can deduce the tilt angle and directional angle immediately. Since what we would like to obtain are the two angles (parameters), we only need two parameters to compute the 1-to-1 mapping operation. We can use wavelength differences, $\left|\lambda_{a}-\lambda_{b}\right|$ and $\left|\lambda_{c}-\lambda_{d}\right|$ as the two parameters. If we properly design the wavelength ranges of the four FBGs to make $\left|\lambda_{a}-\lambda_{b}\right|$ and $\left|\lambda_{c}-\lambda_{d}\right|$ distinct from each other at a certain directional angle, say 0 degrees, due to the different tendencies of the peak shifts, we have non-overlapped data points when using $\left|\lambda_{a}-\lambda_{b}\right|$ and $\left|\lambda_{c}-\lambda_{d}\right|$ to represent the tilt and directional angles. Using the 1-to-1 mapping operation, we can obtain the tilt and directional angles simultaneously in real-time. For example, in the present case, $\left|\lambda_{a}-\lambda_{b}\right|$ = 14.2 nm and $\left|\lambda_{c}-\lambda_{d}\right|$ = 8.1 nm at a 0° tilt angle (initial point, see Figure 6). When tilted to a 0° directional angle at a 2.5° tilt angle, $\left|\lambda_{a}-\lambda_{b}\right|$ = 5.5 nm and $\left|\lambda_{c}-\lambda_{d}\right|$ = 8.1 nm, while when tilted to a 180° directional angle, $\left|\lambda_{a}-\lambda_{b}\right|$ = 22.3 nm and $\left|\lambda_{c}-\lambda_{d}\right|$ = 8.1 nm. From the above data, we can also deduce the resolution of directional angle measurement. At a tilt angle of 2.5°, $\left|\lambda_{a}-\lambda_{b}\right|$ changed from 5.5 nm to 22.3 nm when the directional angle changed from 0° to 180°, and we have a directional angle measurement resolution of 0.00187 rad when using an OSA with a resolution of 0.01 nm. However, the accuracy of the directional angle measurement is inevitably dependent on the tilt angle because the larger the tilt angle, the greater the peak wavelength shifts. 

## 4. Conclusions

In this paper, a temperature-independent highly-sensitive two-dimensional inclinometer based on two crisscross-etched chirped fiber Bragg grating arrays is experimentally demonstrated with a best tilt angle sensing resolution of 0.003°. Due to the unique sensing mechanism, the sensor is temperature insensitive. This sensor is ideal for real-time monitoring of very small tilting in harsh environments. For proof-of-concept, the current data were presented using ether as the liquid medium. Other materials can be used to improve the sensitivity and the robustness. Depending on the applications, it is possible to further improve the dynamic range and sensitivity by optimizing the design parameters of the sensor. For example, increasing the distance between the gratings to increase the dynamic range, or redesign the curvature or shape of the container to increase the interaction length of the grating to increase the sensitivity. This sensor is compact in size and readily compatible with typical inclinometers. We envision this sensor to be useful as a cost-effective field-deployable inclinometer for demanding applications where precision is critical.
